# Lane Endpoint Detection and Position Accuracy Evaluation for Sensor Fusion-Based Vehicle Localization on Highways

**DOI:** 10.3390/s18124389

**Published:** 2018-12-11

**Authors:** Eun Seok Jang, Jae Kyu Suhr, Ho Gi Jung

**Affiliations:** 1 Department of Electronic Engineering, Korea National University of Transportation, 50 Daehak-ro, Chungju-si, Chungbuk 27469, Korea; esjang9919@gmail.com; 2 School of Intelligent Mechatronics Engineering, Sejong University, 209 Neungdong-ro, Gwangjin-gu, Seoul 05006, Korea; jksuhr@sejong.ac.kr

**Keywords:** lane endpoint detection, position accuracy evaluation, vehicle localization, sensor fusion, intelligent vehicle

## Abstract

Landmark-based vehicle localization is a key component of both autonomous driving and advanced driver assistance systems (ADAS). Previously used landmarks in highways such as lane markings lack information on longitudinal positions. To address this problem, lane endpoints can be used as landmarks. This paper proposes two essential components when using lane endpoints as landmarks: lane endpoint detection and its accuracy evaluation. First, it proposes a method to efficiently detect lane endpoints using a monocular forward-looking camera, which is the most widely installed perception sensor. Lane endpoints are detected with a small amount of computation based on the following steps: lane detection, lane endpoint candidate generation, and lane endpoint candidate verification. Second, it proposes a method to reliably measure the position accuracy of the lane endpoints detected from images taken while the camera is moving at high speed. A camera is installed with a mobile mapping system (MMS) in a vehicle, and the position accuracy of the lane endpoints detected by the camera is measured by comparing their positions with ground truths obtained by the MMS. In the experiment, the proposed methods were evaluated and compared with previous methods based on a dataset acquired while driving on 80 km of highway in both daytime and nighttime.

## 1. Introduction

Vehicle localization is one of the key components of both autonomous driving and advanced driver assistance systems (ADAS). Accurate localization results can improve performances of other key components such as perception, planning, and control. The most widely used vehicle localization method is global navigation satellite systems (GNSS). This method provides global positions and its error is not accumulated, but produces inaccurate results when the GNSS signal is reflected or blocked. To alleviate this problem, GNSS has often been fused with dead reckoning (DR). This method is robust against the status of the GNSS signal and provides accurate results in a short period of time, but its error can accumulate over time. Recently, to overcome these drawbacks, localization methods that utilize a perception sensor and digital map have been widely researched [1]. These methods localize the ego-vehicle by matching the landmarks detected by the perception sensor and the landmarks stored in the digital map.

**Figure 1:**
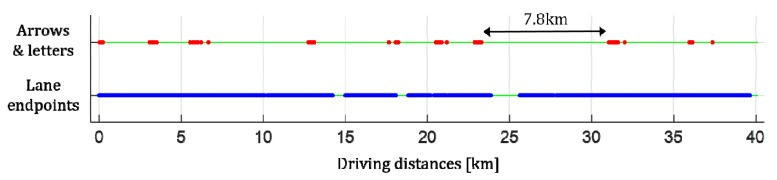
Analysis of distributions of road markings in highway situations.

Various types of landmarks have been used, and road markings are one of the most widely used landmarks for vehicle localization. In terms of the perception sensor, road markings are relatively easy to detect because their shapes are predetermined by regulations and their color and reflectivity are significantly different from those of the road surface. In terms of the digital map, road markings can be stored at low capacity because their shapes are relatively simple compared with other road structures. Among road markings, lane markings are most widely used for vehicle localization. Lane markings provide abundant information on lateral position, but lack information on longitudinal position. To alleviate the drawback of lane markings, methods that utilize stop lines, crosswalks, arrows, and letters have been suggested. Those road markings are useful in urban situations because they frequently appear on urban roads. However, in highway situations, stop lines and crosswalks rarely exist, and arrows and letters seldom appear. Figure 1 shows the results of analyzing sequential images taken from approximately 40 km of highway from the viewpoint of road markings. First of all, this highway does not include any stop lines and crosswalks. In general, there are no stop lines and crosswalks on Korean highways. Red points in Figure 1 indicate the locations where arrows and letters were captured by the front camera. The arrows and letters were observed only in approximately 3% of the entire images. Furthermore, the arrows and letters are concentrated in specific areas such as intersections. The roads where the arrows and letters are not continuously observed reach up to 7.8 km. This analysis clearly shows that stop lines, crosswalks, arrows, and letters have limitations of improving longitudinal positioning accuracy in highway situations.

**Figure 2:**
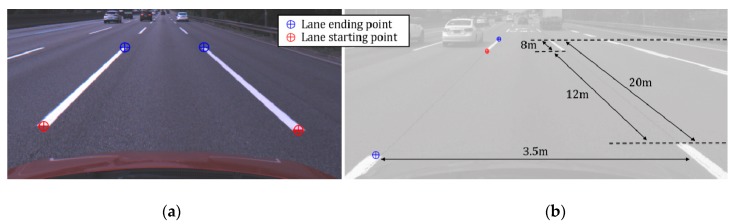
(**a**) Two types of lane endpoints; (**b**) Installation regulation of dashed lane marking.

To overcome the limitations of the abovementioned road markings, lane endpoints can be used as landmarks to improve longitudinal positioning accuracy. The lane endpoint indicates the location where a lane marking starts or ends, so this paper classifies it into a lane starting point and a lane ending point. Figure 2a shows lane endpoints taken in a highway. Red and blue crosses indicate the starting and ending points, respectively. As a landmark for vehicle localization in highway situations, the lane endpoint has the following advantages: (1) Since it represents a specific point, it provides information on both lateral and longitudinal positions; (2) Since it has a simple and distinct shape, it can effectively be detected using a conventional automotive front camera; (3) Since it can be captured at a close distance, its position accuracy is guaranteed; (4) It frequently appears in highway situations. In Figure 1, blue points indicate the locations where the lane endpoints were captured by the front camera while driving on approximately 40 km of highway. The lane endpoints were observed in approximately 88% of the entire images, and the roads where the lane endpoints are not continuously observed reach at most to 1.7 km in a tunnel. According to Korean regulations, the length of a single dashed lane marking is 8.0 m, longitudinal distance between two dashed lane markings is 12.0 m, and the width of the driving lane is at least 3.5 m as shown in Figure 2b. This means that the lane endpoints of the same type (staring or ending point) appear every 20.0 m in the longitudinal direction and every 3.5 m in the lateral direction. Because there is enough space between adjacent lane endpoints, there is little confusion between adjacent lane endpoints when matching them with those stored in the digital map. The effect of using a specific location of a road marking for vehicle localization has been verified in [2] where arrow endpoints are used instead of lane endpoints. Note that this paper suggests that the lane endpoints are used as one of several landmarks, rather than suggesting the use of lane endpoints alone.

This paper proposes two methods concerning the use of the lane endpoints as landmarks for vehicle localization. First, it proposes a method to efficiently detect the lane endpoints using a vehicle-mounted monocular forward-looking (front) camera, which is the most widely installed perception sensor. Second, it proposes a method to reliably measure the position accuracy of the lane endpoints detected from images taken while the ego-vehicle is moving at high speed.

Since the front camera module is responsible for detecting various objects such as lane markings, vehicles, pedestrians, traffic signs, etc., an algorithm added to this module should require less computational costs. Thus, this paper proposes a method that can efficiently detect lane endpoints by combining simple algorithms. The proposed method first extracts lane pixels using a top-hat filter, and detects left and right lanes based on RANSAC. Once the lanes are detected, profiles of the top-hat filter response are generated along the detected lanes. Lane endpoint candidates are generated by finding local minima and maxima from differentiation results of the profiles. The lane endpoint candidates are verified by a two-class classifier after converting them into bird’s-eye view images. Finally, the positions of the lane endpoints from the camera are calculated based on the camera’s intrinsic and extrinsic parameters.

In general, the landmark-based localization system estimates the position of the ego-vehicle by comparing the positions of the detected landmarks from the ego-vehicle and those stored in the digital map. This means the position accuracy of the detected landmark is directly related to the performance of the vehicle localization system. In addition, it is necessary to have a distribution of the position error of the detected landmark in order to effectively apply extended Kalman or particle filtering methods. However, it is quite difficult to precisely measure the position accuracy of the landmarks, which are detected from images taken while the ego-vehicle is moving on a highway at high speed. According to our literature review, there has been no previous work attempting it. To reliably measure the position accuracy of the landmark, this paper proposes a method that uses a mobile mapping system (MMS) after examining various approaches. Since the MMS consists of high-precision positioning equipment and high performance LIDARs, it produces highly accurate positions and dense 3D points with reflectivities. If the front camera is attached to the MMS, 3D locations of the detected lane endpoints from the front camera can be accurately obtained while the ego-vehicle is moving at high speed by using the precise positions and dense 3D points produced by the MMS. Thus, this paper reliably measures the position accuracy of the lane endpoints by comparing its position obtained from the camera and the corresponding position obtained from the MMS.

In the experiment, the proposed method was evaluated based on a dataset acquired while driving on approximately 80 km of highway. Half of the dataset was acquired during daytime and the other half at night. The proposed lane endpoint detection method shows 96.1% recall and 99.7% precision in daytime, and 94.7% recall and 100% precision at night. As a result of the MMS-based position accuracy evaluation, the detected lane endpoints show 7.8 cm and 21.6 cm average position errors in lateral and longitudinal directions during daytime, and 8.2 cm and 48.2 cm average position errors in lateral and longitudinal directions at night. In terms of execution time, this method requires only 4.35 ms per image so that it can handle 230 frames per second.

The rest of this paper is organized as follows: Section 2 explains related research. Section 3 and Section 4 describe the proposed lane endpoint detection method and the MMS-based position accuracy evaluation method, respectively. Section 5 presents experimental results and analyses. Finally, this paper is concluded with future works in Section 6.

## 2. Related Research

**Figure 3:**
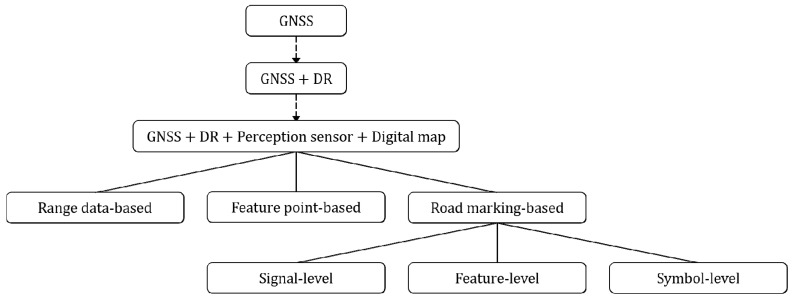
Taxonomy of vehicle localization methods.

The perception sensor and digital map-based vehicle location methods can be categorized based on the information acquired by the perception sensor and stored in the digital map. According to this criterion, previous methods can be categorized into range data-based, feature point-based, and road marking-based approaches. Since the proposed system is categorized into the road marking-based approach, the other two approaches are briefly introduced in this section. Figure 3 shows the taxonomy of the vehicle localization methods.

The range data-based approach acquires range data using perception sensors and matches them with range information stored in the digital map. Since this approach mostly utilizes active sensors such as LIDARs and Radars, it is robust against illumination conditions and textures of surrounding objects. However, its performance can be degraded in cases where a few fixed objects and many moving obstacles are presented on a broad road. The methods in this approach recognize specific objects (e.g., curbs [3], building facades [4], pole-like objects [5], or guardrails [6]), or directly utilize 3D point clouds [7] to match them with the digital map.

The feature point-based approach extracts feature points mostly from images and matches them with feature points stored in the digital map by comparing their descriptors. Because this approach utilizes a large number of feature points on various surrounding objects, it can achieve high localization accuracy. However, its performance can be affected by appearance and position changes of obstacles. In addition, it requires a large storage volume to store numerous feature points along with their high-dimensional descriptors in the digital map. The methods in this approach utilize various types of interest point detectors and feature descriptors: maximally stable extremal regions (MSER) with moment invariants [8], difference of Gaussians (DoG) with scale-invariant feature transform (SIFT), fast Hessian detector with speeded up robust features (SURF) [9], and DIRD (Dird is an illumination robust descriptor) [10].

The road marking-based approach extracts markings on road surfaces and matches them with road markings stored in the digital map. Since road markings are visually distinctive and under government regulations, they can be reliably detected compared to other objects. However, localization performance can be degraded when the road markings are worn or covered with snow. The road marking-based approach can further be categorized into signal-level, feature-level, and symbol-level approaches according to the level of information being used.

The signal-level approach uses raw data of road surfaces obtained from the perception sensor. Mattern et al. [11] matches intensities of image pixels with a virtual road surface image generated from the digital map. Levinson and Thrun [12] compares infrared reflectivities of a LIDAR with their means and standard deviations stored in the digital map. The feature-level approach utilizes features of the road marking extracted from raw data of the perception sensor. Hata and Wolf [13], Kim et al. [14], and Suganuma and Uozumi [15] find road marking features by comparing infrared reflectivities acquired by LIDARs with neighboring reflectivities or threshold values. Schreiber et al. [16] extracts road marking features by applying the oriented matched filter to free space obtained by a stereo camera. Deusch et al. [17] uses the maximally stable extremal regions to find road marking features from images of a front camera. Jo et al. [18] utilizes the top-hat filter to extract road marking features from bird’s-eye view images. The signal-level and feature-level approaches require less effort to handle the outputs of the perception sensors. However, since the raw data and road marking features have a large amount of information, these approaches require a high computational cost for the matching procedure and a large storage volume for the digital map.

The symbol-level approach recognizes a variety of types of road markings and matches them with those stored in the digital map. Lane markings are the most popularly used symbols for vehicle localization. Nedevschi et al. [19], Jo et al. [20], Lu et al. [21], Gruyer et al. [22], Tao et al. [23], Shunsuke et al. [24], and Suhr et al. [2] utilize a variety of types of cameras to detect lane markings for vehicle localization purposes. In particular, Nedevschi et al. [19] not only recognize positions of lanes but also their types (e.g., double, single, interrupted, and merge) as additional information. Even though the lane markings provide abundant information on the lateral position, they lack information on the longitudinal position. To complement this, stop lines, crosswalks, arrows, and letters have been utilized as well. Nedevschi et al. [19] recognizes stop-lines using a stereo camera and Jo et al. [20] recognize crosswalks using a front camera in order to enhance longitudinal localization accuracies. Nedevschi et al. [19] detect five types of arrows using a stereo camera to recognize the driving lane of the ego-vehicle. Wu and Ranganathan [25] recognize ten types of arrows and letters using a stereo camera and extract corner features from them. Suhr et al. [2] recognize nine types of arrows and diamond along with their starting points using a single front camera. The symbol-level approach requires a low computational cost for the matching procedure and a small storage volume for the digital map because it simply matches and stores locations and types of symbols. However, it requires additional computing resources to reliably recognize specific symbols. Thus, the symbol recognition procedure should be designed to be cost-effective.

The lane endpoints are expected to be good landmarks for vehicle localization when applying the symbol-level approach to highway situations. This paper proposes two essential components for its application: a cost-effective method to detect the lane endpoints using a conventional monocular front camera and a reliable method to measure the position accuracy of the lane endpoints based on the MMS. According to our literature review, there has been no previous work that explicitly detects the lane endpoint for vehicle localization purposes and measures its position accuracy.

## 3. Lane Endpoint Detection

### 3.1. Lane Detection

**Figure 4:**
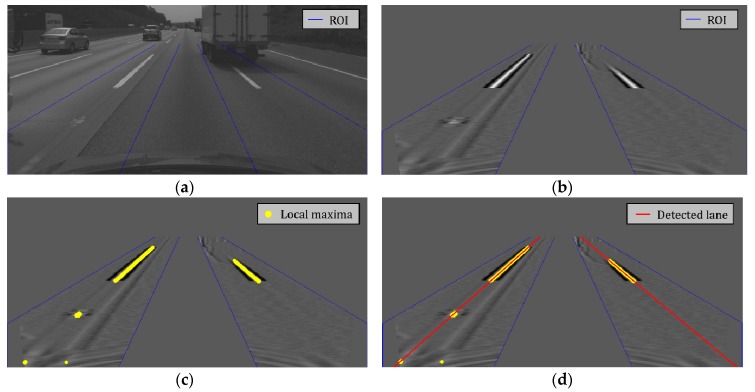
Procedures for lane detection. (**a**) Regions of interest for lane detection; (**b**) Top-hat filtering result; (**c**) Local maxima locations; (**d**) Lane detection result.

For lane departure warning (LDW) and lane keeping assist (LKA) systems, distant lanes and their curvatures should be obtained. However, in this paper, lane detection results are used only to restrict the search range of lane endpoints. For this purpose, it is enough to detect the lanes close to the ego-vehicle. Since the highway has a large turning radius, the lanes close to the ego-vehicle can be approximated as straight lines. Thus, the lanes within 20 m of the front camera are detected by finding a pair of straight lines. To detect left and right lanes, this paper first designates regions of interest (ROIs) for both lanes by considering the camera’s intrinsic and extrinsic parameters and the lane width of the highway in the regulation. Two pairs of blue lines in Figure 4a indicate the ROIs for left and right lanes. Lane candidate pixels are extracted by applying a horizontal top-hat filter to the ROIs shown in Figure 4a [26]. To reduce the computational cost, the top-hat filter is not applied to bird’s-eye view images but to the original images. The horizontal top-hat filter, **h** can be described as

(1) $$h=\left[-1_{w/2},\;1_{w},\;1_{w/2}\right]$$

where 1*_n_* is an *n*-dimensional row vector whose elements are all ones. The top-hat filter can successfully detect lane candidate pixels if *w* fits the lane marking width. The lane marking width changes according to the vertical coordinate of the image because of the perspective projection. Thus, this paper pre-calculates the lane marking widths at different vertical coordinates based on the camera’s intrinsic and extrinsic parameters and the width of the lane marking in the regulation. In this pre-calculation, it is assumed that the roll and yaw angles of the camera are close to zero and only markings of the ego-lane are considered. The response of the top-hat filter at location (*u*,*v*), *r*(*u*,*v*) can be cost-efficiently calculated using only four operations regardless of *w* when a horizontal integral image, *I_h_* is used as:

(2) $$r\left(u,v\right)=2\left[I_{h}\left(u+w/2,v\right)-I_{h}\left(u-w/2,v\right)\right]-\left[I_{h}\left(u+w,v\right)-I_{h}\left(u-w,v\right)\right]$$

Figure 4b shows the result of applying the top-hat filter to the ROIs in Figure 4a. Once the top-hat filter response is obtained, local maxima are searched for each row of the image to find the center pixels of the lane markings. Yellow points in Figure 4c indicate the locations of the extracted local maxima whose top-hat filter responses are larger than a predetermined value. Finally, a pair of lines is estimated by separately applying the RANSAC-based line detector to the local maxima found from the left and right ROIs [27]. Two red lines in Figure 4d show the detected left and right lanes. The lane markings are considered to be stably detected if the vanishing point calculated by a pair of two lines is located at a similar position more than *N* (in this paper, =3) consecutive images. Two consecutive vanishing points are considered to be located in a similar position if the distance between them is less than a predetermined value. Once the lane markings are stably detected, the ROIs for left and right lanes are limited to the narrow areas near the lane markings detected in the previous image. Even though this paper detects lanes using a combination of simple algorithms to reduce computational cost, more sophisticated methods can also be used.

### 3.2. Lane Endpoint Candidate Generation

**Figure 5:**
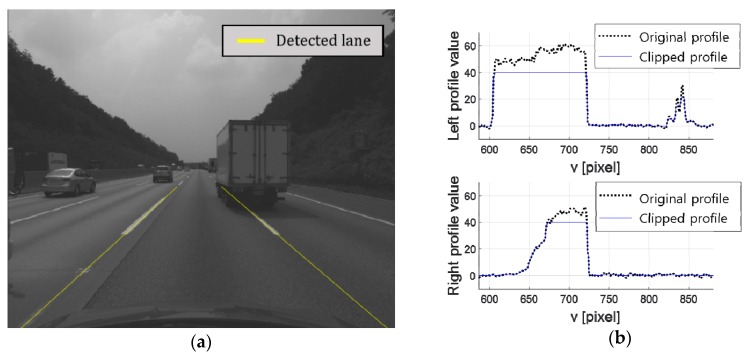

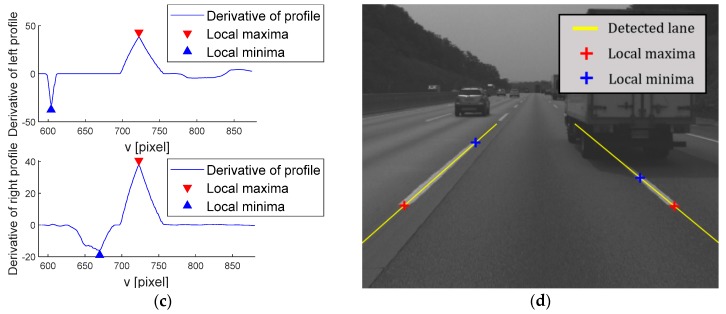
Lane endpoint candidate generation. (**a**) Lane detection result; (**b**) Left and right lane profiles; (**c**) Derivatives of the lane profiles and candidates of the lane starting and ending points; (**d**) Candidates of the lane starting and ending points in the image.

The lane endpoint candidates are generated along the detected lanes. To this end, lane profiles are first calculated by following the detected left and right lanes. Those profiles are not generated based on the brightness values of the image, but the response of the top-hat filter already obtained in the lane detection procedure. Direct use of the brightness values is sensitive to lighting conditions because even objects of the same color in the real world can have different brightness values in images depending on local lighting conditions. However, since the response of the top-hat filter is calculated based on the brightness difference between surrounding areas, it is relatively robust against lighting conditions. The lane profile, **p** can be represented as:

(3) p=[p(N), p(N+1), … , p(M−1), p(M)]

where *p*(*y*) indicates the value of the lane profile at the vertical image location, *y*. *N* and *M* are the starting and ending locations of the lane profile, and they correspond to the road surface of 5 m and 20 m in front of the front camera, respectively. *p*(*y*) is calculated from the top-hat filter response as:

(4) $$p\left(y\right)=\max\left\{r\left(L_{x}\left(y\right)-1,y\right),\;r\left(L_{x}\left(y\right),y\right),\;r\left(L_{x}\left(y\right)+1,y\right)\right\}$$

where *r*(*x*,*y*) is the value of the top-hat filter response at the image location (*x*,*y*), and *L*_x_(*y*) is the horizontal image location of the lane marking at *y*. *p*(*y*) is calculated not only using *r*(*L_x_*(*y*),*y*) but also using its horizontally neighboring values, *r*(*L_x_*(*y*) − 1,*y*) and *r*(*L_x_*(*y*) + 1,*y*) in order to reduce the influence of the lane detection error on the generation of the lane profile. Figure 5a shows an example of the lane detection result, and black dotted lines in Figure 5b show the lane profiles extracted from the left and right lane markings detected in Figure 5a. It can be seen that the lane profiles have large values at lane marking locations. The extracted lane profiles are clipped to make their maximum and minimum values become predetermined values, and filtered by the median filter to alleviate the noise. Blue solid lines in Figure 5b show the lane profiles after the clipping and filtering. Once the lane profile is extracted and preprocessed, it is differentiated to find the lane endpoints. The differential value at *y*, *d*(*y*) is calculated by subtracting the averages of the lane profile values before and after 1 m from *y* as:

(5) $$d\left(y\right)=\left\{\frac{1}{y-m_{y}^{-1}}\sum_{i=m_{y}^{-1}}^{y-1}p\left(i\right)\right\}-\left\{\frac{1}{m_{y}^{+1}-y}\sum_{i=y+1}^{i=m_{y}^{+1}}p\left(i\right)\right\}$$

where *m_y_*^−1^ and *m_y_*^+1^ are the vertical image locations before and after 1 m from *y*, respectively. Figure 5c shows the derivatives of the lane profiles. In the derivative, a peak indicates the location where the lane profile value abruptly increases, which is the starting point of the lane, and a valley is the location where the lane profile value abruptly decreases, which is the ending point of the lane. Thus, local maxima and minima whose absolute values exceed a predetermined value in the derivatives are extracted and designated as candidates of the starting and ending points. To prevent multiple starting or ending points from being generated at similar locations, the non-maxima (or non-minima) suppression is conducted.

Red and blue triangles in Figure 5c show the generated candidates of the starting and ending points, and red and blue crosses in Figure 5d show the corresponding locations in the image. Since the proposed method detects the lane endpoint based on the lane marking detection result, the lane endpoints cannot be detected when both the left and right lane markings are not stably detected.

### 3.3. Lane Endpoint Candidate Verification

In real situations, the candidates of the lane endpoints (including starting and ending points) may include some false detections due to preceding vehicles, stains, shadows, etc. From the viewpoint of the vehicle localization, the false detections can more severely degrade the localization accuracy than the miss detections. If landmarks are miss-detected, the matching between the map and landmarks is omitted and the vehicle localization is performed with DR, which slightly degrades the localization accuracy. However, if landmarks are falsely detected, the localization accuracy may be significantly degraded due to the matching of the map with the wrong landmarks [2]. Therefore, this paper verifies the generated lane endpoint candidates in order to eliminate the falsely generated candidates.

**Figure 6:**
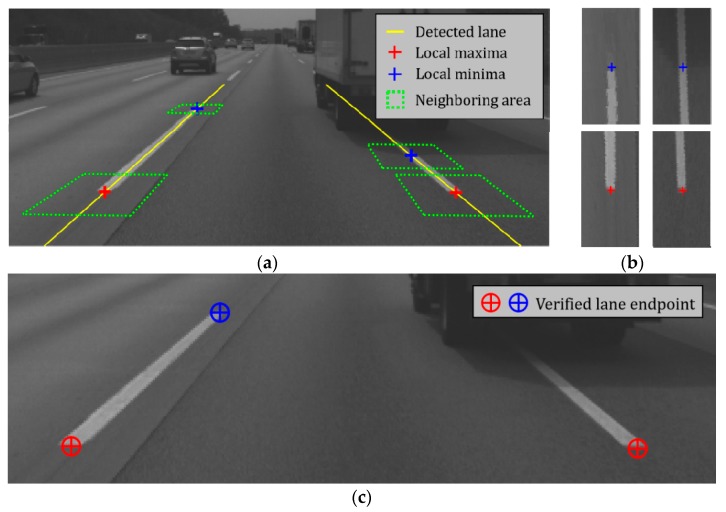
Lane endpoint candidate verification. (**a**) Lane endpoint candidates; (**b**) Bird’s-eye view images generated from lane endpoint candidates; (**c**) Lane endpoint verification result.

To this end, the area of 1 m × 2 m around the lane endpoint candidate is first transformed into a bird’s-eye view image. In this transformation, the vertical direction of the bird’s-eye view image is set to coincide with the direction of the detected lane. Crosses in Figure 6a are four lane endpoint candidates generated from the procedure shown in Figure 5, and green rectangles are 1 m × 2 m areas around the candidates. Figure 6b shows four bird’s-eye view images generated from the green rectangles in Figure 6a. Since this transformation removes the perspective distortion, it can increase the verification performance by reducing the inter-class variation of the correctly generated candidates. After the transformation, the feature extraction and classification are conducted based on the bird’s-eye view image to determine whether the candidate is correct. The histogram of oriented gradients (HOG) [28] and support vector machine (SVM) [29] are used for the feature extraction and classification, respectively. The HOG-SVM is a long-proven method and has been widely used for various classification tasks such as vehicle, pedestrian, traffic sign, etc. It has an advantage that a high classification performance can be achieved with a small amount of computation when applied to a problem in which the degree of difficulty is not high. In this paper, the HOG-SVM is selected after comparing it with the convolutional neural network (CNN), which is a class of deep learning, which is known to provide the highest classification performance in image classification [30]. Both methods provided similar classification performance, but the HOG-SVM is far ahead in terms of computational cost. This will be explained in detail in the experimental section.

The resolution of the bird’s-eye view image is 48 × 96 pixels, and the parameters of the HOG are as follows: number of orientation bin: 9; cell size: 8 × 8 pixels; block size: 2 × 2 cells; and block overlapping: 50%. Thus, the HOG feature dimension is 1980. After extracting the HOG feature, the SVM is used to determine whether the candidate is correct. In order to reduce the computational cost, the linear SVM is selected. In general, the classification function of the SVM is defined as:

(6) $$f\left(x\right)=\sum_{i}^{N}\alpha_{i}y_{i}\Phi {\left(x_{i}\right)}^{T}\Phi \left(x\right)+b$$

where **x***_i_* is the *i*-th support vector and *N* is the number of support vectors. *α_i_* is a coefficient of **x***_i_*, and *b* is a bias. **x** is an input feature vector and *y_i_* indicates the class of **x***_i_*, which is either −1 or +1. Φ is a kernel function. In case of the linear SVM, (6) can be simplified as:

(7) $$\begin{array}{l} f\left(x\right)=\sum_{i}^{N}\alpha_{i}y_{i}x_{i}^{T}x+b=w^{T}x+b,\\ w=\sum_{i}^{N}\alpha_{i}y_{i}x_{i} \end{array}$$

Since *α_i_*, *y_i_*, and **x***_i_* can all be determined at the learning stage, **w** can be calculated in advance. Therefore, the test stage can be simplified by adding the bias, *b* to the inner product of two vectors, **w** and **x** regardless of the number of support vectors. This simplification significantly reduces the computational cost. If a lane endpoint candidate is classified as a correct one based on the HOG-SVM classifier, it is confirmed as a final detection result. Details of the learning and evaluation of the HOG-SVM classifier will be described in the experimental section. Figure 6c shows the lane endpoint verification result. It can be seen that one falsely generated lane endpoint candidate is correctly removed during the lane endpoint verification procedure.

### 3.4. Calculation of Lane Endpoint Location

To effectively utilize the detected lane endpoints for vehicle localization, their locations with respect to the camera should be calculated. This paper calculates the location of the lane endpoint based on the camera’s intrinsic and extrinsic parameters, which are obtained during the precalibration stage using planar checkerboards. In case of calibrating the extrinsic parameters, the longitudinal direction of the vehicle is set to the Y-axis, the ground to the XY-plane, and the direction perpendicular to the ground to Z-axis in the checkerboard coordinate system. If R*_B2C_* and **t***_B2C_* are rotation matrix and translation vector that represent a rigid transformation between the checkerboard and camera coordinate systems, an image location, (*x*,*y*) and a 2D location in the XY-plane of the checkerboard coordinate system, (*X_B_*,*Y_B_*) are related by a homography, H:

(8) $$\begin{array}{l} H=K\left[\begin{array}{ccc} r_{1} & r_{2} & t_{B2C} \end{array}\right],\\ R_{B2C}=[\begin{array}{ccc} r_{1} & r_{2} & r_{3} \end{array}] \end{array}$$

Since the origin of the 2D location of the lane endpoint should be the origin of the camera, H in (8) is manipulated as:

(9) $$\begin{array}{l} H^{\prime }=K\left[\begin{array}{ccc} r_{1} & r_{2} & {t^{\prime }}_{B2C} \end{array}\right],\\ {t^{\prime }}_{B2C}=r_{3}r_{3}^{T}t_{B2C} \end{array}$$

Based on (9), the 2D location of the lane endpoint having the camera location as its origin, (*X_C_*, *Z_C_*) can be calculated from the image location, (*x*,*y*) using the homography, H’ as:

(10) $$\begin{array}{l} X_{C}=\frac{a}{c},\;Z_{C}=\frac{b}{c},\\ {\left[\begin{array}{ccc} a & b & c \end{array}\right]}^{T}={H^{\prime }}^{-1}{\left[\begin{array}{ccc} x & y & 1 \end{array}\right]}^{T} \end{array}$$

*X_C_* and *Z_C_* are called the lateral and longitudinal locations of the lane endpoint, respectively.

## 4. Position Accuracy Evaluation

### 4.1. Candidates for Evaluation Methods

Unfortunately, after extensive literature review, we have not found any previous methods for evaluating the position accuracy of the road markings, which are detected from images taken during high-speed driving. Thus, this section introduces the methods considered as candidates for position accuracy evaluation, and explains the reason why the MMS-based method is finally selected. The following three methods have been considered as candidates: measuring instrument-based, on-vehicle sensor-based, and MMS-based.

The measuring instrument-based method measures the relative position from the camera to the road marking using various measuring devices. The terrestrial laser scanning (TLS) is one of the most widely used instruments, and is mainly used for obtaining high resolution 3D models of large structures such as dams, mines, and terrain. To evaluate the position accuracy of the road marking using this method, TLS and the monocular front camera are first installed together and the rigid transformation between them should be precalibrated. The ground truth position of the road marking is obtained from 3D points produced by the TLS, and its position is transformed into the camera coordinate system using the rigid transformation between the TLS and camera. Finally, the position accuracy is measured by comparing the transformed ground truth position and the position calculated by the image of the camera. This method has an advantage of providing a dense and precise 3D shape of the road marking. However, it can be used only in cases where the vehicle is stationary because the TLS takes a long time to scan surrounding areas. For the same reason, it is difficult for the TLS to scan long and narrow areas such as highways. Due to these limitations, it has been concluded that this method is inappropriate to use in highway situations.

The on-vehicle sensor-based method measures the relative position from the camera to the road marking using various ranging sensors that have already been mounted on commercialized or autonomous vehicles. The most widely used ranging sensors mounted on those vehicles are a stereo camera and multi-layer LIDAR. This method is similar to the measuring instrument-based method. However, there is a major difference, which is the sensing speed. The sensing speed of the vehicle mounted ranging sensors are designed to be fast so that they can quickly obtain the ranging data of the surrounding area while the vehicle is moving at high speeds. To evaluate the position accuracy of the road marking using this method, the stereo camera or LIDAR along with the monocular front camera are first installed on the same vehicle and the rigid transformation between those sensors should be precalibrated. The ground truth position of the road marking is obtained from a dense disparity map of the stereo camera or 3D points produced by the multi-layer LIDAR, and its position is transformed into the camera coordinate system using the rigid transformation between those sensors. Finally, the position accuracy is measured by comparing the transformed ground truth position and the position calculated by the image of the camera. This method has an advantage that it is applicable to situations where the vehicle is moving at high speeds. However, in the case of the stereo camera, its ranging accuracy severely deteriorates when the distance of the road marking increases. In the case of the multi-layer LIDAR, it provides a high ranging accuracy, but its range data is not dense enough to accurately localize the road marking. Due to these limitations, it has been concluded that this method is inappropriate to use.

The MMS-based method measures the relative position from the camera to the road marking using dense 3D points and accurate positions obtained by the MMS. The MMS consists of high-precision positioning equipment and high performance LIDARs, and those sensors are usually mounted on a vehicle. Since the MMS can measure dense and accurate 3D points while the vehicle is moving at high speeds, it has an advantage of measuring long and wide areas such as highways. To evaluate the position accuracy of the road marking using this method, the monocular front camera is first attached to the MMS and the rigid transformation between the camera and MMS should be precalibrated. The ground truth position of the road marking is obtained from 3D points and its position is transformed into the camera coordinate system using the precalibrated rigid transformation and accurate positions produced by the MMS. Finally, the position accuracy is measured by comparing the transformed ground truth position and the position calculated by the image of the camera. Compared to the measuring instrument-based method, the MMS-based method has an advantage that it can be used while the vehicle is moving at high speeds. Compared to the on-vehicle sensor-based method, its 3D structure is denser and more accurate. Because of these advantages, this paper has selected the MMS-based method for evaluating the position accuracy of the road marking. A detailed explanation of this method will be presented in the following sections.

### 4.2. Acquisition of 3D Points Using MMS

The MMS used in this paper consists of a high precision LIDAR as a ranging sensor and a combination of real-time kinematic-global positioning system (RTK-GPS), high precision inertial measurement unit (IMU), and distance measurement instrument (DMI) as a positioning sensor. Table 1 summarizes the specifications of these sensors. 

**Figure 7:**
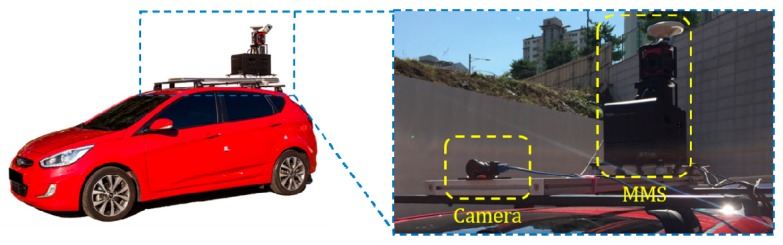
MMS and camera used in the experiment.

**Figure 8:**
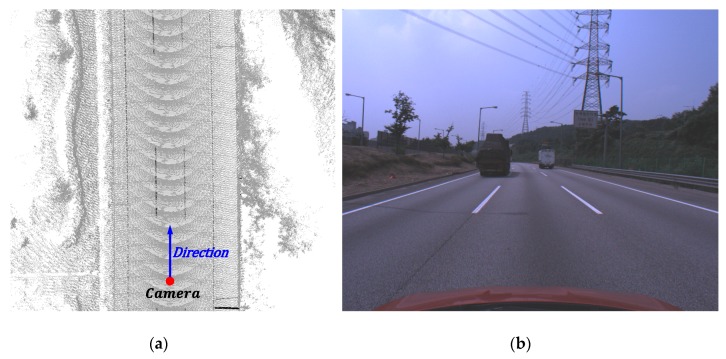
Example 3D structure and image captured by MMS and camera. (**a**) 3D structure obtained by the MMS; (**b**) Image captured by the camera. A red point and blue arrow in (**a**) indicate the camera position at the time when the image (**b**) was captured.

The mapping and positioning sensors are mounted on an off-the-shelf vehicle as shown in Figure 7. 3D structures surrounding the vehicle is obtained by registering 3D points acquired by the ranging sensor using 6D positions produced by the positioning sensor while the vehicle is moving. The obtained 3D points include 3D locations and infrared reflectivities. The monocular camera used to detect the lane endpoints is mounted on the vehicle equipped with the MMS, and the rigid transformation between the camera and MMS is precalibrated. Since an image is captured by being triggered by the positioning sensor, the precise camera position at the time of capturing the image are stored. Figure 8a,b show an example 3D structure and image captured by the MMS and camera, respectively. The red point and blue arrow in Figure 8a indicate the camera position at the time when the image in Figure 8b is captured.

### 4.3. Acquisition of Lane Endpoint Ground Truth

To evaluate the position accuracy of the lane endpoint, the ground truth position of the lane endpoint is manually designated using the 3D points obtained by the MMS. Since the manually designated position is in the 3D point cloud coordinate system, it is converted into the camera coordinate system using the precise camera position at the time of capturing the image, which is provided by the MMS. The position accuracy is evaluated by comparing the ground truth position and the position produced by the proposed method.

**Figure 9:**
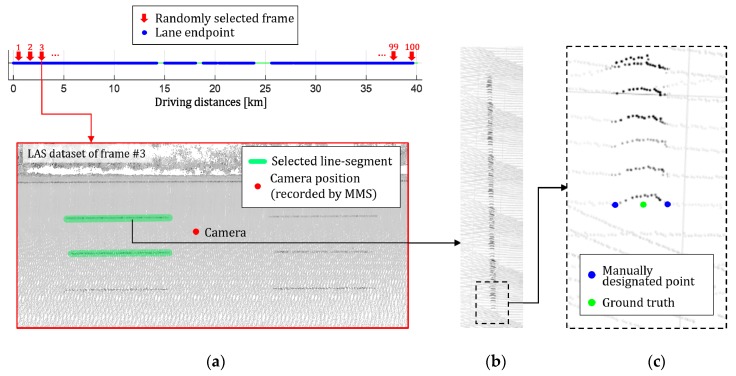
Ground truth acquisition procedure. (**a**) 3D points around the selected camera position; (**b**) Enlargement of dashed lane segment; (**c**) Lane endpoint ground truth designation.

To manually designate the lane endpoints in the 3D points, roads and lanes should be visually distinguishable. However, since the roads and lanes are located on the same plane, it is hard to distinguish between them using their 3D information. Thus, this paper utilizes the infrared reflectivities provided by the LIDAR of the MMS to visually distinguish the roads and lanes. The 3D points of the lanes have high infrared reflectivities compared with the roads because they are drawn with a highly reflective paint. The positions of the lane endpoints are manually designated by displaying the 3D points based on their infrared reflectivities. Figure 9a shows the displayed 3D points based on their infrared reflectivities. In this figure, the larger the infrared reflectivity, the darker the 3D point is drawn. It can be noticed that the lanes are darker than the roads. This paper randomly selected 100 camera positions from approximately 40 km of highway, and manually designated four lane endpoints from the closest pair of dashed lanes. Figure 9a shows the 3D points around a randomly chosen camera position, which is depicted by a red point. In this figure, two green dashed lines indicate the selected dashed lane pair, which is closest to the camera position. Figure 9b shows an enlargement of one of the two selected lanes. Since the proposed method detects the center of the lane end as shown in Figure 5, the same location should be designated in the 3D points. To this end, the left and right corners of the lane end are manually designated and their center location is used as the ground truth of the lane endpoint. Figure 9c shows an example of the lane endpoint designation. In this figure, two blue points indicate two manually designated locations and a green point indicates the center of the two blue points, which is the ground truth of the lane endpoint.

### 4.4. Evaluation of Lane Endpoint Position Accuracy

Since the position of the lane endpoint is calculated with respect to the camera position in Section 3.4, its ground truth position obtained in Section 4.3 should be transformed into the camera coordinate system. To this end, the manually designated ground truth, **G***_P_* in the point cloud coordinate system is first transformed into the camera coordinate system, **G***_C_* as:

(11) $$G_{C}=R_{P2C}\left(G_{P}-t_{P2C}\right)$$

where R*_P_*_2*C*_ and **t***_P_*_2*C*_ are the rotation matrix and translation vector that represent a rigid transformation between the point cloud and camera coordinate systems. R*_P_*_2*C*_ and **t***_P_*_2*C*_ are obtained from the high precision positioning sensor of the MMS. **G***_C_* is manipulated as:

(12) $${\left[\begin{array}{ccc} X_{G} & Y_{G} & Z_{G} \end{array}\right]}^{T}={\left(R_{B2C}\right)}^{T}\left(G_{C}-{t^{\prime }}_{B2C}\right),$$

where R*_B_*_2*C*_ and **t**′*_B_*_2*C*_ are the rotation matrix and the translation vector that represent a rigid transformation between the camera and checkerboard coordinate systems, which has been already explained in (8) and (9). *X_G_* and *Z_G_* are the ground truth position of the lane endpoint in lateral and longitudinal directions, respectively. The position accuracy of the lane endpoint is calculated by comparing (*X_G_* and *Z_G_*) in (12) and (*X_C_* and *Z_C_*) in (10).

## 5. Experiments

### 5.1. Experimental Environment

**Figure 10:**
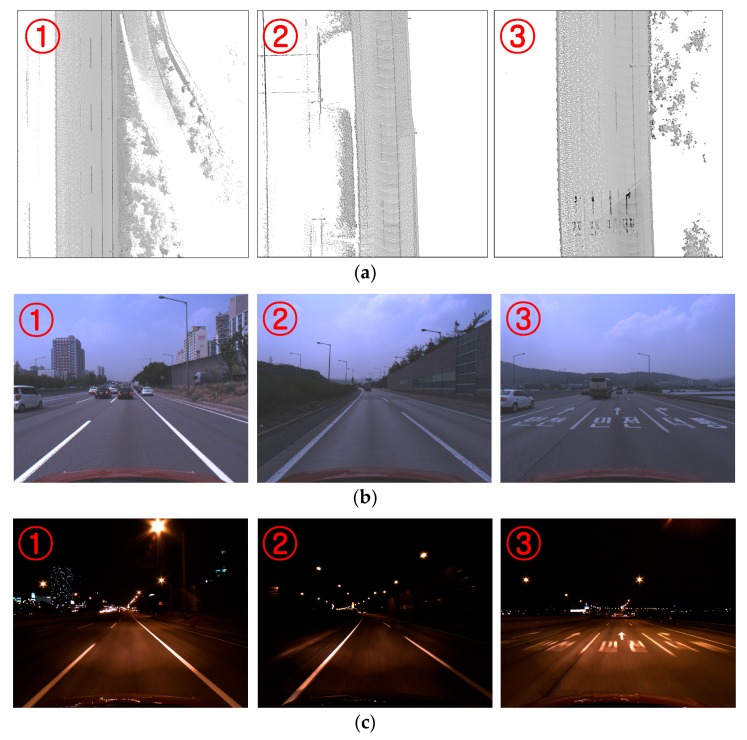
Highway the vehicle drove when acquiring the test dataset. (**a**) 3D points at three sample locations; (**b**) Images taken in daytime; (**c**) Images taken at nighttime.

Experiments were conducted using a dataset acquired by the monocular forward-looking (front) camera and the MMS mounted on the roof of the vehicle while driving on highways during the day and night. The experimental setup has been shown in Figure 7. The resolution, horizontal field of view, and acquisition frequency of the front camera are 1280 × 1024 pixels, 60 degrees, and 20 Hz, respectively. The dataset was acquired while driving at approximately 70~80 km/hour on average. It includes a total 80 km of driving. Half of the dataset was taken during daytime (13:00 p.m.) and half at night (21:00 p.m.). Figure 10a shows the 3D points of three sample locations (①, ②, and ③) included in the test dataset. Figure 10b,c show images taken by the front camera at those three locations in daytime and nighttime, respectively.

### 5.2. Performance Evaluation and Comparison of Lane Endpoint Verification

**Figure 11:**
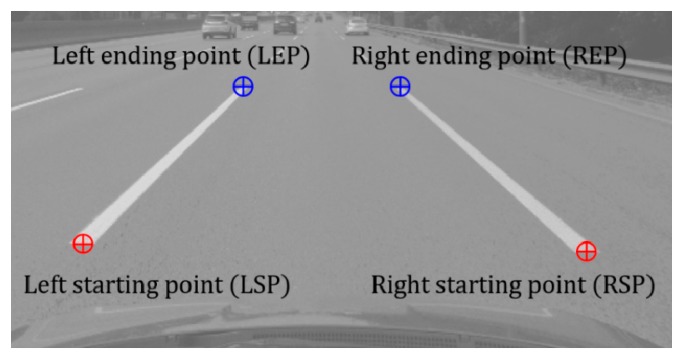
Types of lane endpoints.

**Figure 12:**
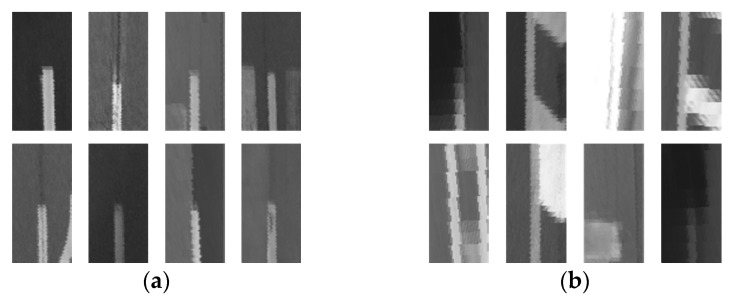
Sample images, (**a**) positive samples; (**b**) negative samples.

This section presents the performance evaluation and comparison results of the lane endpoint verification explained in Section 3.3. To train and test the lane endpoint verifier, this paper collected training and test samples from images that are totally different from the test dataset introduced in Section 5.1. The collected sample images were manually divided into positive and negative samples. The lane endpoints are categorized into four types: left staring point (LSP), left ending point (LEP), right starting point (RSP), and right ending point (REP) as shown in Figure 11. This paper trains one classifier for each type of the lane endpoint so that a total of four classifiers are trained. Table 2 shows the number of sample images used for training and testing the lane endpoint verifiers. Figure 12 shows positive and negative samples images.

This paper compares two methods: the HOG-SVM and CNN. The CNN-based classifier has been widely used for a variety of applications. This paper chose the one that has been used for classifying road markings in [30] for performance comparison. The performances of the two methods were evaluated based on the following three criteria: true positive rate (TPR), true negative rate (TNR), and classification accuracy. The better the classification performance, the greater the value of all three criteria:

(13) $$\begin{array}{l} \mathrm{TPR}=\frac{\mathrm{No}.\;\mathrm{of}\;\mathrm{correctly}\;\mathrm{classified}\;\mathrm{positive}\;\mathrm{samples}}{\mathrm{No}.\;\mathrm{of}\;\mathrm{positive}\;\mathrm{samples}}\\ \mathrm{TNR}=\frac{\mathrm{No}.\;\mathrm{of}\;\mathrm{correctly}\;\mathrm{classified}\;\mathrm{negative}\;\mathrm{samples}}{\mathrm{No}.\;\mathrm{of}\;\mathrm{negative}\;\mathrm{samples}}\\ \mathrm{Accuracy}=\frac{\mathrm{No}.\;\mathrm{of}\;\mathrm{correctly}\;\mathrm{classified}\;\mathrm{samples}}{\mathrm{No}.\;\mathrm{of}\;\mathrm{all}\;\mathrm{samples}} \end{array}$$

Two classifiers were trained by the same training samples and tested by the same test samples shown in Table 2. Table 3 and Table 4 show the performances of the HOG-SVM and CNN in [30], respectively. The HOG-SVM gives 98.2% TPR, 98.1% TNR, and 98.1% accuracy in Table 3, and the CNN gives 99.5% TPR, 97.4% TNR, and 98.5% accuracy. In terms of the classification performance, two methods are quite similar. However, in terms of the computational cost, the HOG-SVM requires 0.25 ms while the CNN requires 3.10 ms to classify a single lane endpoint. This means that if four lane endpoint candidates are generated, only the verification stage requires 12.40 ms in the case of using the CNN. Considering that the total computation time of the proposed method is 4.35 ms, the computation time of the CNN is too heavy as a part of the proposed method. Based on this performance evaluation, it is found that the HOG-SVM is more cost-effective than the CNN in this application. The computation time of the proposed method and specification of the used computer will be explained in Section 5.5.

### 5.3. Performance Evaluation of Lane Endpoint Detection

This section presents the performance evaluation and comparison results of the lane endpoint detection, which consists of the lane endpoint candidate generation and verification. To this end, this paper randomly selected 1200 images from the test dataset. Of these, 600 images are from the dataset taken in daytime, and 600 images at night. The daytime and nighttime images include 1504 and 1290 lane endpoints, respectively. This paper manually confirms whether the locations of the detected lane endpoints match the location of the actual lane endpoints. The performance evaluation and comparison were conducted based on the following three criteria: recall, precision, and F-measure. The better the classification performance, the greater the value of all three criteria:

(14) $$\begin{array}{l} \mathrm{Recall}=\frac{\mathrm{No}.\;\mathrm{of}\;\mathrm{true}\;\mathrm{positives}}{\mathrm{No}.\;\mathrm{of}\;\mathrm{lane}\;\mathrm{endpoints}}\\ \mathrm{Precision}=\frac{\mathrm{No}.\;\mathrm{of}\;\mathrm{true}\;\mathrm{positives}}{\mathrm{No}.\;\mathrm{of}\;\mathrm{true}\;\mathrm{positives}\;+\;\mathrm{No}.\;\mathrm{of}\;\mathrm{false}\;\mathrm{positives}}\\ F-\mathrm{measure}=2\times \frac{\mathrm{precision}\;\times \;\mathrm{recall}}{\mathrm{precision}\;+\;\mathrm{recall}} \end{array}$$

**Figure 13:**
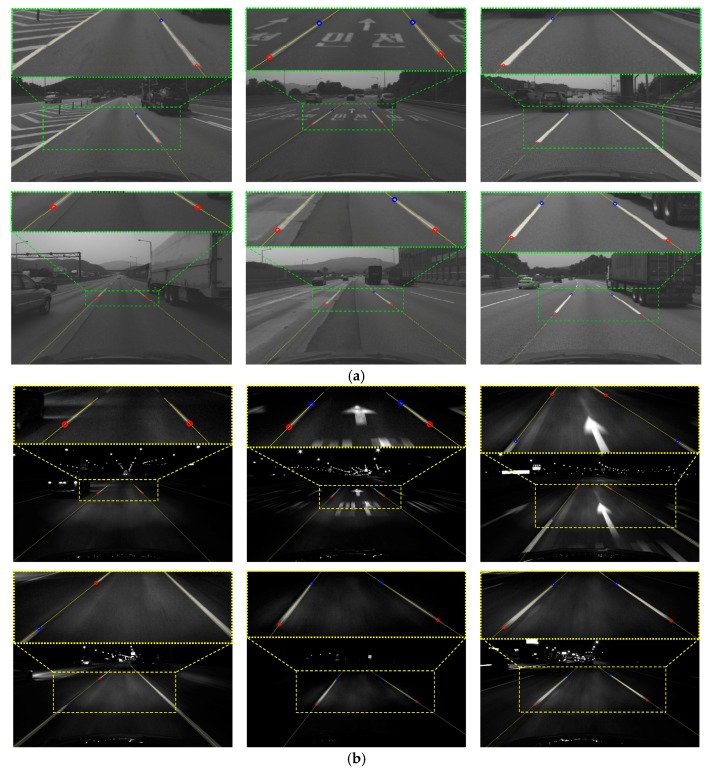
Lane endpoint detection results of the proposed method in (**a**) daytime and (**b**) nighttime.

Table 5 and Table 6 show the performances of the proposed method in daytime and nighttime, respectively. In the case of the daytime performance, this method gives 96.1% recall by correctly detecting 1445 lane endpoints out of 1504, and gives 99.7% precision by producing only four false detections. The F-measure that combines recall and precision is 97.9%. In the case of the nighttime, this method gives 94.7% recall by correctly detecting 1222 lane endpoints out of 1290, and gives 100.0% precision by producing no false detection. The F-measure is 97.3%. These results show that the proposed method successfully detects lane endpoints both in daytime and nighttime and produces very few false detections. Figure 13a,b show the lane endpoint detection results of the proposed method in daytime and nighttime, respectively. As aforementioned in Section 3.3, from a vehicle localization perspective, it is a great advantage that the proposed method generates very few false positives because falsely detected landmarks can cause a large localization error by being mismatched with the landmarks stored in the digital map. As shown in Table 5 and Table 6, the recall of the nighttime performance is slightly lower than that of the daytime. This is because in daytime, illumination is relatively uniform depending on the distance, but at night, the area at near and far distance from the vehicle is dark due to lack of headlight lighting as shown in Figure 13b and this slightly hinders detection. The precision of the nighttime is slightly higher than that of the daytime. This is because objects in front of the ego-vehicle have low brightness values in images at night as shown in Figure 13b, which reduces the possibility of producing false detections.

The proposed method was quantitatively compared with the method suggested in [31]. The method in [31] detects the lane endpoint for camera calibration purposes. Table 7 and Table 8 show the performance of the method suggested in [31] in daytime and nighttime, respectively. In the case of the daytime performance, this method gives 97.1% recall and 66.3% precision. In the case of the nighttime performance, it gives 88.3% recall and 60.4% precision. Compared with the proposed method, the method in [31] shows a similar recall, but there is a large difference (30%~40%) in precision. This difference is mainly caused by two reasons. One reason is because of the approach of measuring the image brightness difference when detecting the lane endpoints. The proposed method measures the brightness difference using neighboring pixels while adaptively changing the size of the top-hat filter. However, the method in [31] measures the brightness difference using distant pixels in the areas of fixed size. Due to the perspective distortion in images of the front camera, it is advantageous to adaptively change the size of the area used to calculate the brightness difference. In addition, it is preferred to use the neighboring pixels when calculating the brightness difference because other road marking such as arrows or letters can degrade the detection performance if the distant pixels are used. The other reason for the large difference in precision is the use of the learning-based lane endpoint verifier. The method in [31] detects the lane endpoints using the image brightness difference and the heuristically tuned threshold values. However, the proposed method utilizes the image brightness difference along with the lane endpoint verifier trained based on a machine learning approach. These are two main reasons why the proposed method produces a higher precision than the method in [31].

### 5.4. Position Accuracy Evaluation of Lane Endpoint

The position accuracy of the lane endpoint was evaluated using the MMS-based method explained in Section 4. To this end, the ground truths of the lane endpoints were first acquired from the 3D points. This paper randomly selected 100 locations and manually designated four endpoints for each location. Thus, the total number of ground truths is 400. The lane endpoints automatically detected from the front camera images based on the proposed method were compared with the corresponding ground truths as explained in Section 4.4. Since the camera takes the same lane endpoint multiple times while the ego-vehicle is moving, each ground truth is detected in multiple images. In this experiment, 400 ground truths correspond with 3829 lane endpoints detected in the daytime images and 4962 lane endpoints detected in the nighttime images. Thus, a total of 8791 lane endpoints detected by the proposed method were used for the position accuracy evaluation. This paper utilizes three criteria for evaluating the position accuracy: longitudinal error (*e_lon_*), lateral error (*e_lat_*), and Euclidean error (*e_euc_*) as:

(15) $$\begin{array}{l} e_{lon}=\left|Z_{G}-Z_{C}\right|\\ e_{lat}=\left|X_{G}-X_{C}\right|\\ e_{euc}=\sqrt{{\left(X_{G}-X_{C}\right)}^{2}+{\left(Z_{G}-Z_{C}\right)}^{2}} \end{array}$$

where (*X_G_*, *Z_G_*) and (*X_C_*, *Z_C_*) are the locations of the ground truth and detected lane endpoints, respectively. *e_lon_* indicates the position error in the vehicle traveling direction, *e_lat_* indicates the position error in the direction perpendicular to the vehicle traveling direction, and *e_euc_* indicates the Euclidean distance between two positions.

**Figure 14:**
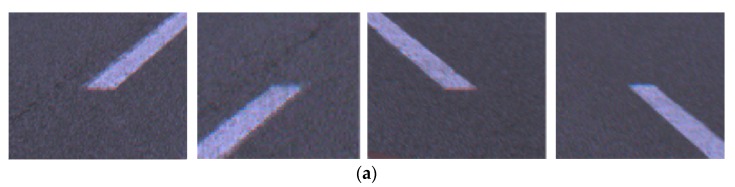

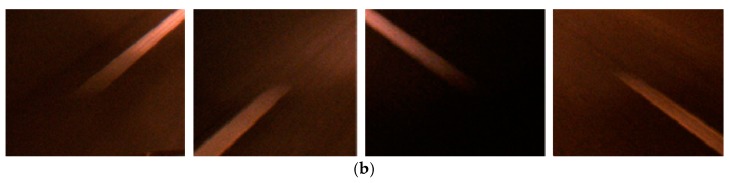
Images of lane endpoints in (**a**) daytime and (**b**) nighttime.

Table 9 shows the mean and standard deviation of the position errors for four types of lane endpoints in daytime and nighttime. In daytime, the proposed method gives 21.6 cm, 7.8 cm, and 24.2 cm for *e_lon_*, *e_lat_*, and *e_euc_*, respectively. In nighttime, the proposed method gives 48.2 cm, 8.2 cm, and 49.9 cm for *e_lon_*, *e_lat_*, and *e_euc_*, respectively. In the case of the highway situation, the lane width is more than 3.5 m and the distance to the preceding vehicle is several tens of meters. Therefore, it can be said that the longitudinal and lateral errors of the lane endpoints detected by the proposed method are sufficient to be used as landmarks of the vehicle localization system for highway autonomous driving. According to Table 9, in both daytime and nighttime, the longitudinal error is much larger than the lateral error. This is because, in the case of using the front camera whose optical axis is almost parallel to the road surface, the number of pixels per meter decreases more sharply in the longitudinal direction compared with the lateral direction. In this table, it can be also found that the longitudinal error of the nighttime (48.2 cm) is much larger than that of the daytime (21.6 cm). This is because the lane endpoints are clearly captured in daytime due to bright illumination but at night, severe motion blurs occur near the lane endpoints due to dark illumination. Figure 14a,b show images of the lane endpoints taken in daytime and nighttime, respectively. It can be clearly noticed that the images taken at nighttime severely contaminated by the motion blur compared with those taken in daytime. Unlike the longitudinal errors, the lateral errors measured in daytime and nighttime are quite similar. This is because the motion blur occurs mainly in the moving direction of the ego-vehicle and hardly occurs in the direction perpendicular to it.

Furthermore, at night, the longitudinal errors of the LEP and REP are larger than those of the LSP and RSP by approximately 20 cm. This is due to the nature of the motion blur that occurs at the lane endpoints. When the front camera is moving at high speed and capturing images, the motion blur makes the brightness of the lane starting point (LSP or RSP) slightly dark. Since the lane marking is much brighter than surrounding areas, a slight darkening does not affect the locations of the detected lane starting points. However, in the same situation, the motion blur makes the brightness of the region near the lane ending point (LEP or REP) fairly bright. This makes the proposed method erroneously detect the location brightened by the motion blur and it leads to a relatively larger longitudinal error.

**Figure 15:**
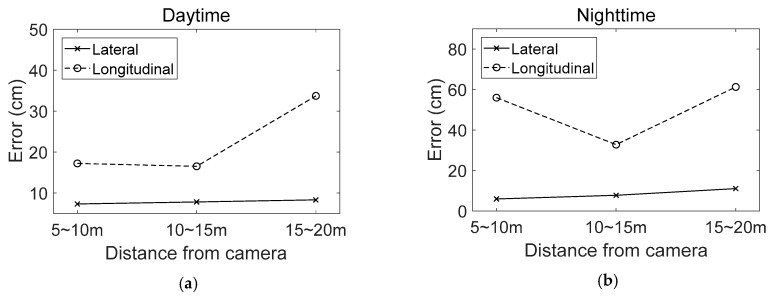
Changes of position errors according to the distance from the camera, (**a**) Daytime; (**b**) Nighttime.

Figure 15 shows the changes in average position errors according to the distance from the camera to the lane endpoint. In Figure 15a, solid and dashed lines indicate the lateral and longitudinal errors in daytime, respectively. In daytime, the lateral error slightly increases and the longitudinal error dramatically increases while the distance to the lane endpoint increases. This is because, in the case of the front camera whose optical axis is almost parallel to the road surface, the number of pixels per meter slightly decreases in the lateral direction and rapidly decreases in the longitudinal direction. 

In Figure 15b, solid and dashed lines indicate the lateral and longitudinal errors at night, respectively. Similar to the daytime case, the lateral error slightly increases while the distance to the lane endpoint increases. However, the longitudinal error shows a tendency different from that of the daytime. The longitudinal error becomes large at the location near the front camera. This is because the motion blur more severely occurs on images of objects that are closer to the camera. Objects that are closer to the camera move faster in a captured image compared with those that are far from the camera.

### 5.5. Execution Time

Table 10 shows the execution times of the main modules of the proposed method. These times were measured on an Intel Core i7-7700 CPU with 16 GB RAM using only a single core. The proposed method requires a total execution time of 4.35 ms, which means that it can process 230 frames per second in real time. Since the proposed method requires only a tiny amount of computation cost, it is possible to be inserted as an additional function of the existing multi-functional front camera module. Note that the image acquisition time is not included in the execution time.

## 6. Conclusions and Future Works

To increase the accuracy of landmark-based vehicle localization, this paper proposes using lane endpoints as landmarks. Two methods are proposed regarding the lane endpoints. One is to efficiently detect the lane endpoints using a conventional front camera, and the other is to reliably measure the position accuracy of the detected lane endpoints. In the experiment, it was found that the proposed detection method can accurately find the lane endpoints in both daytime and nighttime with a small amount of computation and the proposed MMS-based method can reliably measure the position accuracy of the lane endpoints detected from images taken while the vehicle is moving at high speed. In the future, we are planning to develop a method that can reduce the influence of the motion blur when calculating the positions of the lane endpoints at night, and extend the proposed position accuracy evaluation method to other landmarks such as arrows, letters, and traffic signs.

## Figures and Tables

**Table 1:** Sensor specifications of the MMS.

Sensor	Model	Specification	
Ranging sensor	Velodyne HDL32E	Measurement range	5 cm~100 m
		Field of view	Horizontal 360° Vertical 40°
		Accuracy	<2 cm
		Angular resolution	Vertical 1.25°
		Acquisition frequency	5~20 Hz
Positioning sensor	Applanix POS LV 210	X, Y position	0.020 m (RMS)
		Z position	0.050 m (RMS)
		Roll & Pitch	0.020° (RMS)
		Heading	0.050° (RMS)
		Acquisition frequency	100 Hz

**Table 2:** Number of samples used for training and testing the lane endpoint verifiers.

	Training Sample		Test Sample	
	Positive	Negative	Positive	Negative
LSP	5000	5202	5000	4582
LEP	5000	5202	5000	4582
RSP	5000	5202	5000	4582
REP	5000	5202	5000	4582

**Table 3:** Performance of the HOG-SVM-based lane endpoint verifier.

	No. of Positive Samples	No. of Negative Samples	No. of True Positives	No. of True Negatives	True Positives Rate	True Negative Rate	Accuracy
LSP	5000	4582	4810	4490	96.2%	98.0%	97.1%
LEP	5000	4582	4905	4408	98.1%	96.2%	97.2%
RSP	5000	4582	4955	4559	99.1%	99.5%	99.3%
REP	5000	4582	4960	4531	99.2%	98.9%	99.1%
**Overall**	**20,000**	**18,328**	**19,630**	**17,988**	**98.2%**	**98.1%**	**98.1%**

**Table 4:** Performance of the CNN-based lane endpoint verifier.

	No. of Positive Samples	No. of Negative Samples	No. of True Positives	No. of True Negatives	True Positives Rate	True Negative Rate	Accuracy
LSP	5000	4582	4961	4368	99.2%	95.3%	97.4%
LEP	5000	4582	4978	4469	99.6%	97.5%	98.6%
RSP	5000	4582	4974	4485	99.5%	97.9%	98.7%
REP	5000	4582	4984	4527	99.7%	98.8%	99.3%
**Overall**	**20,000**	**18,328**	**19,897**	**17,849**	**99.5%**	**97.4%**	**98.5%**

**Table 5:** Performance of the proposed lane endpoint detection method in daytime.

	No. of End-Points	No. of True Positive	No. of False Positive	Recall	Precision	F-Measure
LSP	364	353	1	97.0%	99.7%	98.3%
LEP	360	334	1	92.8%	99.7%	96.1%
RSP	393	383	0	97.5%	100.0%	98.7%
REP	387	375	2	96.9%	99.5%	98.2%
**Overall**	**1504**	**1445**	**4**	**96.1%**	**99.7%**	**97.9%**

**Table 6:** Performance of the proposed lane endpoint detection method in nighttime.

	No. of End-Points	No. of True Positive	No. of False Positive	Recall	Precision	F-Measure
LSP	355	335	0	94.4%	100.0%	97.1%
LEP	385	362	0	94.0%	100.0%	96.9%
RSP	250	235	0	94.0%	100.0%	96.9%
REP	300	290	0	96.7%	100.0%	98.3%
**Overall**	**1290**	**1222**	**0**	**94.7%**	**100.0%**	**97.3%**

**Table 7:** Performance of the method [31] in daytime.

	No. of End-Points	No. of True Positive	No. of False Positive	Recall	Precision	F-Measure
LSP	364	345	204	94.8%	62.8%	75.6%
LEP	360	343	193	95.3%	64.0%	76.6%
RSP	393	391	208	99.5%	65.3%	78.8%
REP	387	381	138	98.4%	73.4%	84.1%
**Overall**	**1504**	**1460**	**743**	**97.1%**	**66.3%**	**78.8%**

**Table 8:** Performance of the method [31] in nighttime.

	No. of End-Points	No. of True Positive	No. of False Positive	Recall	Precision	F-Measure
LSP	355	322	165	90.7%	66.1%	76.5%
LEP	385	323	214	83.9%	60.1%	70.1%
RSP	250	232	157	92.8%	59.6%	72.6%
REP	300	262	210	87.3%	55.5%	67.9%
**Overall**	**1290**	**1139**	**746**	**88.3%**	**60.4%**	**71.7%**

**Table 9:** Position Accuracy Evaluation Result in Daytime and Nighttime. [Mean (Standard Deviation)].

Type	Daytime			Nighttime		
	*e_lon_* (cm)	*e_lat_* (cm)	*e_euc_* (cm)	*e_lon_* (cm)	*e_lat_* (cm)	*e_euc_* (cm)
LSP	20.9 (21.1)	6.9 (4.1)	23.5 (19.8)	36.3 (34.9)	8.5 (6.2)	38.2 (34.4)
LEP	22.0 (20.6)	7.8 (4.5)	24.7 (19.5)	55.9 (35.2)	8.1 (6.2	57.4 (34.3)
RSP	20.9 (18.0)	8.4 (5.5)	23.7 (17.2)	38.7 (29.5)	8.2 (5.9)	40.6 (28.7)
REP	22.5 (18.3)	8.0 (5.1)	25.0 (17.5)	59.4 (33.8)	8.2 (5.9)	60.9 (32.6)
**Overall**	**21.6 (19.5)**	**7.8 (4.9)**	**24.2 (18.5)**	**48.2 (34.9)**	**8.2 (6.1)**	**49.9 (34.0)**

**Table 10:** Execution time.

Module	Time (ms)
Integral image generation	1.51
Top-hat filtering	1.46
RANSAC-based lane detection	0.35
Lane endpoint candidate generation	0.08
Lane endpoint candidate verification	0.95
Overall	4.35
